# Ex Vivo Plasma Application on Human Brain Microvascular Endothelial-like Cells for Blood–Brain Barrier Modeling

**DOI:** 10.3390/ijms26073334

**Published:** 2025-04-03

**Authors:** Sophie-Charlotte Nelz, Elisabeth Lück, Anne Schölzel, Martin Sauer, Jacqueline Heskamp, Sandra Doss

**Affiliations:** 1 Division of Nephrology, Center for Internal Medicine, Rostock University Medical Center, 18057 Rostock, Germany; sophie-charlotte.nelz@med.uni-rostock.de (S.-C.N.);; 2 Department of Extracorporeal Therapy Systems, Fraunhofer Institute for Cell Therapy and Immunology IZI, 18057 Rostock, Germany; 3 Center for Anesthesiology and Intensive Care Medicine, Hospital of Magdeburg, 39130 Magdeburg, Germany

**Keywords:** microvascular endothelial cells, ex vivo, plasma, blood–brain barrier, in vitro model, hiPSC, differentiation, cell culture medium, physiology

## Abstract

hiPSC-derived blood–brain barrier (BBB) models are valuable for pharmacological and physiological studies, yet their translational potential is limited due to insufficient cell phenotypes and the neglection of the complex environment of the BBB. This study evaluates the plasma compatibility with hiPSC-derived microvascular endothelial-like cells to enhance the translational potential of in vitro BBB models. Therefore, plasma samples (sodium/lithium heparin, citrate, EDTA) and serum from healthy donors were tested on hiPSC-derived microvascular endothelial-like cells at concentrations of 100%, 75%, and 50%. After 24 h, cell viability parameters were assessed. The impact of heparin-anticoagulated plasmas was further evaluated regarding barrier function and endothelial phenotype of differentiated endothelial-like cells. Finally, sodium-heparin plasma was tested in an isogenic triple-culture BBB model with continuous TEER measurements for 72 h. Only the application of heparin-anticoagulated plasmas did not significantly alter viability parameters compared to medium. Furthermore, heparin plasmas improved barrier function without increasing cell density and induced a von Willebrand factor signal. Finally, continuous TEER measurements of the triple-culture model confirmed the positive impact of sodium-heparin plasma on barrier function. Consequently, heparin-anticoagulated plasmas were proven to be compatible with hiPSC-derived microvascular endothelial-like cells. Thereby, the translational potential of BBB models can be substantially improved in the future.

## 1. Introduction

The blood–brain barrier (BBB) is a highly complex barrier located in the capillaries of the central nervous system (CNS) [1]. By its multicellular structure with specialized brain microvascular endothelial cells (BMECs), pericytes, and astrocytes, it limits the diffusion of potentially toxic molecules from the blood compartment, while allowing the transport of important nutrients and water into the brain compartment, at the same time [2]. Under pathological conditions, high accumulation of inflammatory stimuli potentially leads to BBB breakdown, inducing long-term neurological damage to the CNS [3,4]. Consequently, the BBB plays a central role in protecting and maintaining homeostasis of the CNS [2], thus making it highly interesting for basic and pharmacological research [1].

However, the aforementioned mechanisms depend on a variety of biological processes and intercellular communication, which hamper research by their complexity [2]. Therefore, in vivo models were used in the past in order to reproduce this complexity and allow scientific explorations on the BBB in physiological and pathological conditions [5,6]. Yet, the translational potential towards human physiology is limited, as tight-junction proteins like Zonula Occludens-1 (ZO-1) and transporter proteins like P-gp are differentially expressed between humans and rodents [7,8,9]. As a consequence, human in vitro models became more important over time. First, immortalized cell lines like the human cerebral microvascular endothelial cell line hCMEC/D3 were used for establishing in vitro BBB models. Although these cell lines provided good reproducibility and a consistent endothelial phenotype, they showed poor capability of forming a barrier and again led to doubts regarding physiological comparability [10,11]. In order to solve this problem, human induced pluripotent stem cells (hiPSCs) gained interest in recent years as an ethically unproblematic source of stem cells for the differentiation of BBB cell types [11,12]. This way, not only a more physiological, barrier-forming phenotype of endothelial cells was supposed to be established, but also the possibility to create isogenic cocultures of the three BBB cell types [12,13]. However, the differentiation to physiologically accurate BMECs is still a challenge and widely discussed in the literature, as many differentiation protocols fail to induce essential BMEC markers like vascular endothelial cadherin (VECad) and von Willebrand factor (vWF), although showing good barrier functionality [14,15,16].

Besides the necessity for optimization of differentiation protocols to further improve the physiological phenotype of differentiated cells, the translational potential, especially for pathological conditions, remains a challenge. As the BBB divides two compartments (blood and CNS), in vitro models need to mirror the layer between two complex media [2]. The blood compartment especially involves a complex mixture of molecules, proteins, and cells [17]. However, this complexity is often neglected in in vitro models, leading to a strong reduction in physiological comparability [18]. Especially for pathological conditions, this issue needs to be addressed, as spiked cell culture media cannot reflect the composition of pathological blood or plasma [19].

As a consequence, the present study aims at evaluating the compatibility of hiPSC-derived BMEC-like cells with plasma and serum from healthy human donors. This way, the use of undiluted plasma in in vitro BBB models was supposed to be established, which allows a higher physiological comparability.

## 2. Results

### 2.1. Differentiated BMEC-like Cells Lack Essential Endothelial Cell Markers

**Figure 1:**
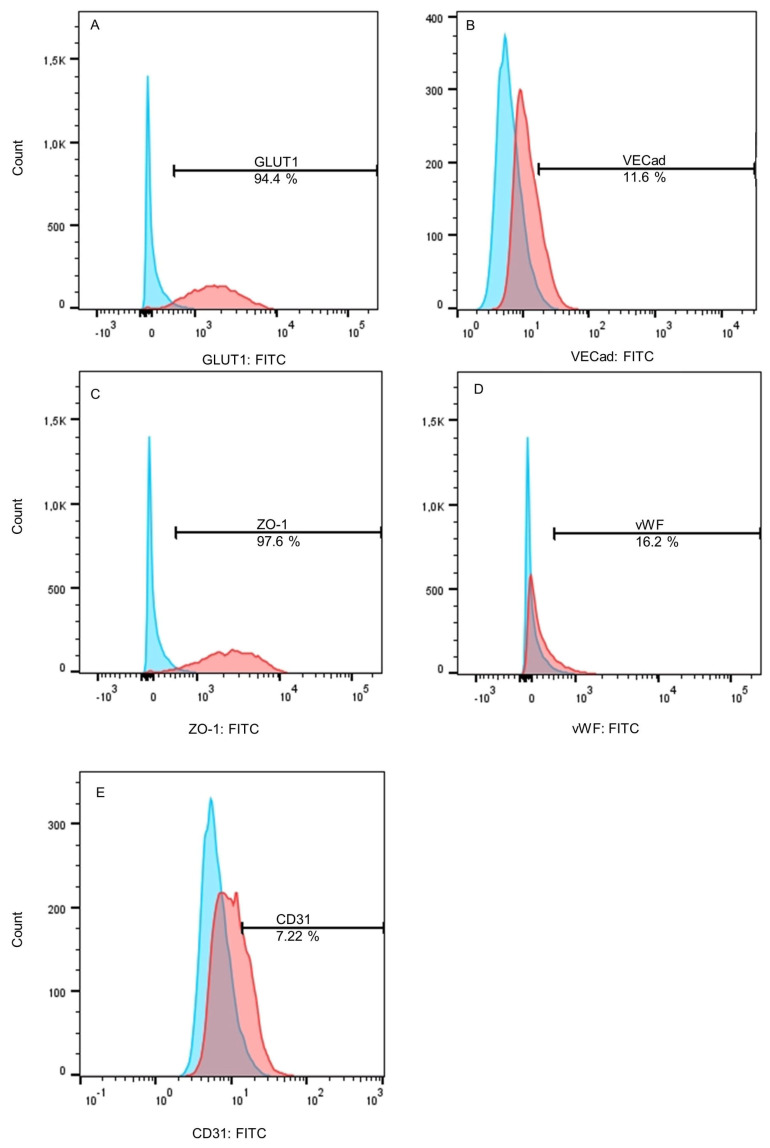
Evaluation of endothelial cell markers (**A**) GLUT1, (**B**) VECad, (**C**) ZO-1, (**D**) vWF, and (**E**) CD31 after differentiation of hiPSC into BMEC-like cells. BMEC-like cells were cultured in serum-free ECM −/−. Secondary antibody or isotype control is depicted in blue and stained samples in red. N = 3.

After differentiation, the endothelial cell markers GLUT1, VECad, ZO-1, vWF, and CD31 were quantified by FACS analysis to evaluate endothelial characteristics. Here, high expressions of GLUT1 and ZO-1 could be shown with about 95% of cells being counted as positive. On the other hand, VECad and vWF expressions were only induced in about 11.6% and 16% of the cells. Additionally, 7.22% of CD31-positive cells could be detected (Figure 1).

**Figure 2:**
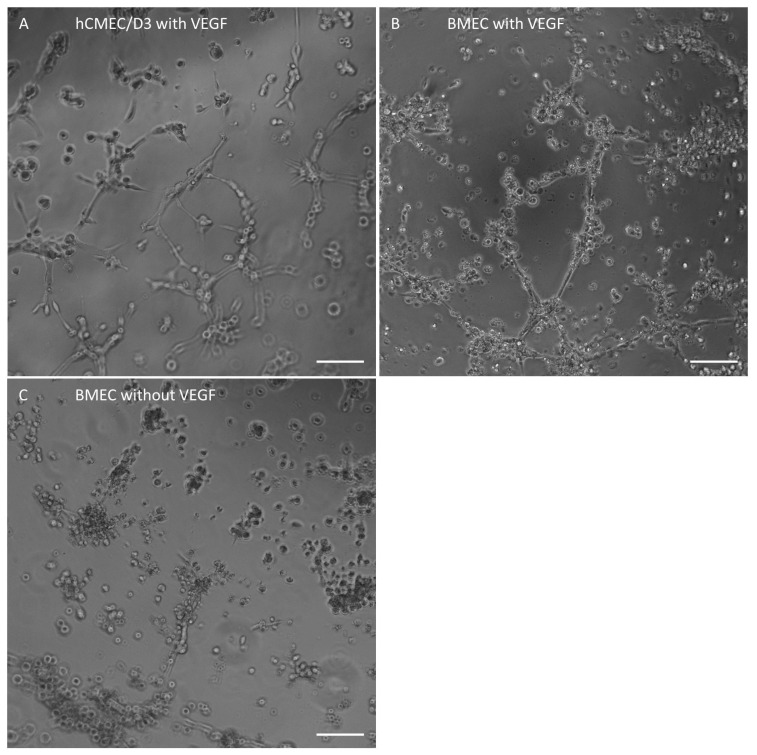
Immortalized hCMEC/D3 (positive control) (**A**) and hiPSC-derived BMEC-like cells with (**B**) and without (**C**) 40 ng/mL VEGF supplementation in ECGM (hCMEC/D3) or serum-free ECM +/+ (BMEC) after 3 h of incubation to induce endothelial tube formation. Scale bar, 100 µm.

Afterwards, an endothelial tube formation assay was conducted. After 3 h of incubation with VEGF, BMEC-like cells showed signs of self-organization and angiogenesis like the immortalized cell line hCMEC/D3. Without VEGF supplementation, BMEC-like cells failed to form these structures (Figure 2).

### 2.2. Undiluted Li-H and So-H Plasmas Can Be Applied on BMEC-like Cells Without Altering Viability

**Figure 3:**
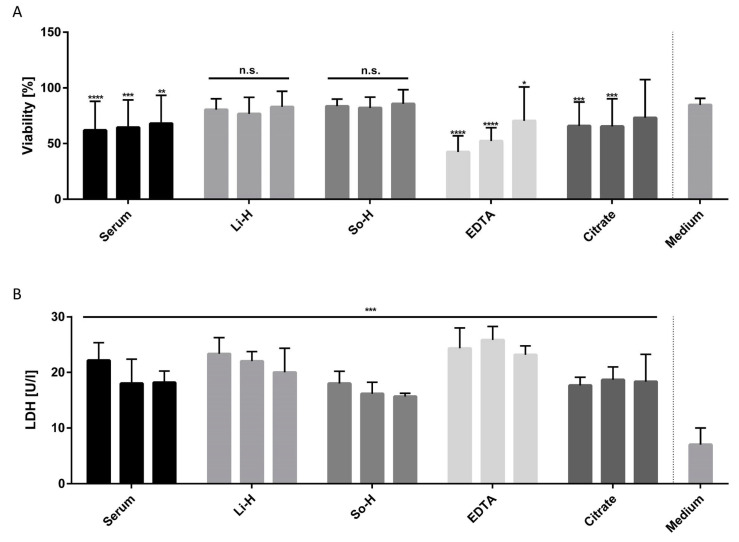
(**A**) Viability and (**B**) LDH secretion after 24 h of incubation with 100% (left), 75% (middle), and 50% (right) plasma/serum diluted in serum-free BMEC medium (ECM −/−). N = 4 for viability and N = 3 for LDH. Statistics: two-way ANOVA with post hoc Dunnett’s multiple comparisons test. **** *p* < 0.0001, *** *p* < 0.001, ** *p* < 0.01, and * *p* < 0.05, n.s. = not significant compared to medium control group.

Next, we examined the compatibility of different anticoagulated plasmas and serum with BMEC-like cells. All plasmas and serum except heparin-anticoagulated plasmas led to significant deterioration of viability in concentrations of 100% and 75%. Additionally, Li-H and So-H anticoagulated plasmas showed no concentration-dependent effects and reached comparable viability values to the medium treated BMEC-like cells (84.67 ± 5.92%), even at a 100% concentration (83.57 ± 6.40% for So-H and 80.49 ± 9.66% for Li-H). Regarding lactate-dehydrogenase (LDH), every plasma- and serum-treated group led to significantly increased secretion compared to the medium (7.00 ± 3.00 U/L), whereas the EDTA-plasma-treated group exhibited some of the highest values with up to 25.83 ± 2.47 U/L LDH. Contrarily, So-H plasma induced the lowest LDH secretion across all treatment groups, peaking at 18.00 ± 2.18 U/L at a 100% concentration (Figure 3). Consequently, undiluted Li-H and especially So-H plasmas are proven to be fundamentally compatible with BMEC-like cells, allowing the use of undiluted plasma in the cell culture.

**Figure 4:**
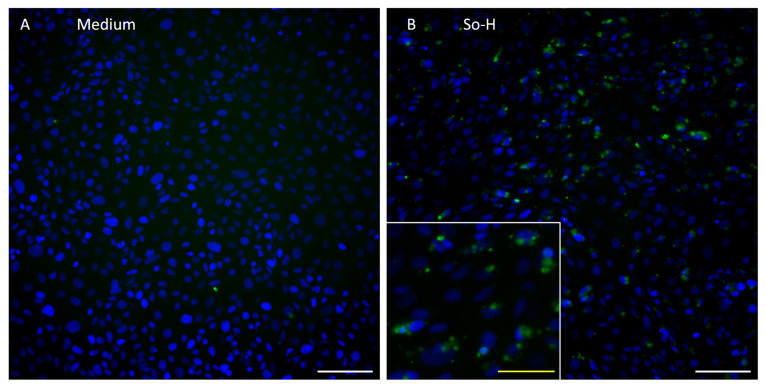
vWF staining of (**A**) serum-free medium (ECM −/−) and (**B**) undiluted So-H plasma-treated BMEC-like cells. vWF (green) and cell nuclei (blue). White scale bar, 100 µm; yellow scale bar, 50 µm.

The expression of the endothelial cell marker vWF in BMEC-like cells was evaluated in medium- and plasma-treated cells. As shown in Figure 4, no fluorescence signal could be detected for the medium-cultured BMEC-like cells, whereas So-H-cultured cells showed positive vWF staining located near the nucleus.

### 2.3. Barrier Properties Improve After Application of Undiluted So-H and Li-H Plasmas Without Significantly Altering Cell Density

**Figure 5:**
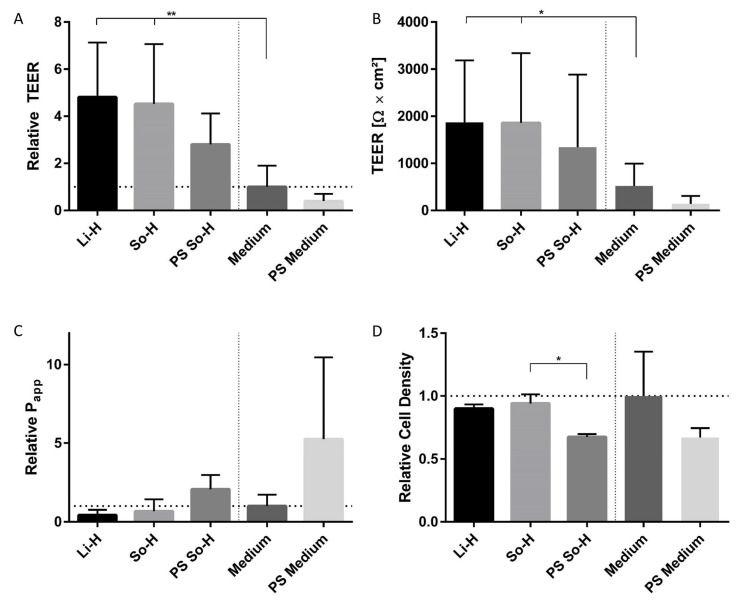
(**A**) Relative and (**B**) absolute TEER, (**C**) P_app_, and (**D**) cell density of BMEC-like cells treated with 100% plasma or serum-free medium (ECM −/−). N = 8 for TEER except PS So-H (N = 5) and N = 5 for P_app_ except PS So-H (N = 3). N = 3 for relative cell density. The horizontal dashed line indicates the value 1, which is used as a reference value for relative data. Statistics: paired *t*-test, * *p* < 0.05, ** *p* < 0.01.

To further analyze the impact of heparin-anticoagulated plasmas on BMEC-like cells, we examined the barrier properties. So-H and Li-H significantly induced more than 4.5 times higher TEER values than the medium control group (521.7 ± 472.4 Ω × cm^2^). Additionally, to demonstrate that the ability of BMEC-like cells to respond to pathological stimulation (PS) is retained in plasma, a combination of cytokines and LPS was used. However, the PS had to be applied at 10 times the concentration in plasma than in medium to reduce TEER in a ratio nearly identical to that observed in medium. These results could be confirmed by FITC-dextran permeability assessment, where the diffusion velocity was nearly halved by the application of Li-H (0.43 ± 0.33) and So-H plasma (0.68 ± 0.75) compared to the medium. In addition to that, cell density measurements were performed to investigate if the higher barrier properties originated from improved proliferation during plasma treatment. Regarding this, no significant differences between the plasma- and medium-treated groups could be detected. Only for the PS So-H-treated group was a significant decrease in cell density measured (Figure 5).

**Figure 6:**
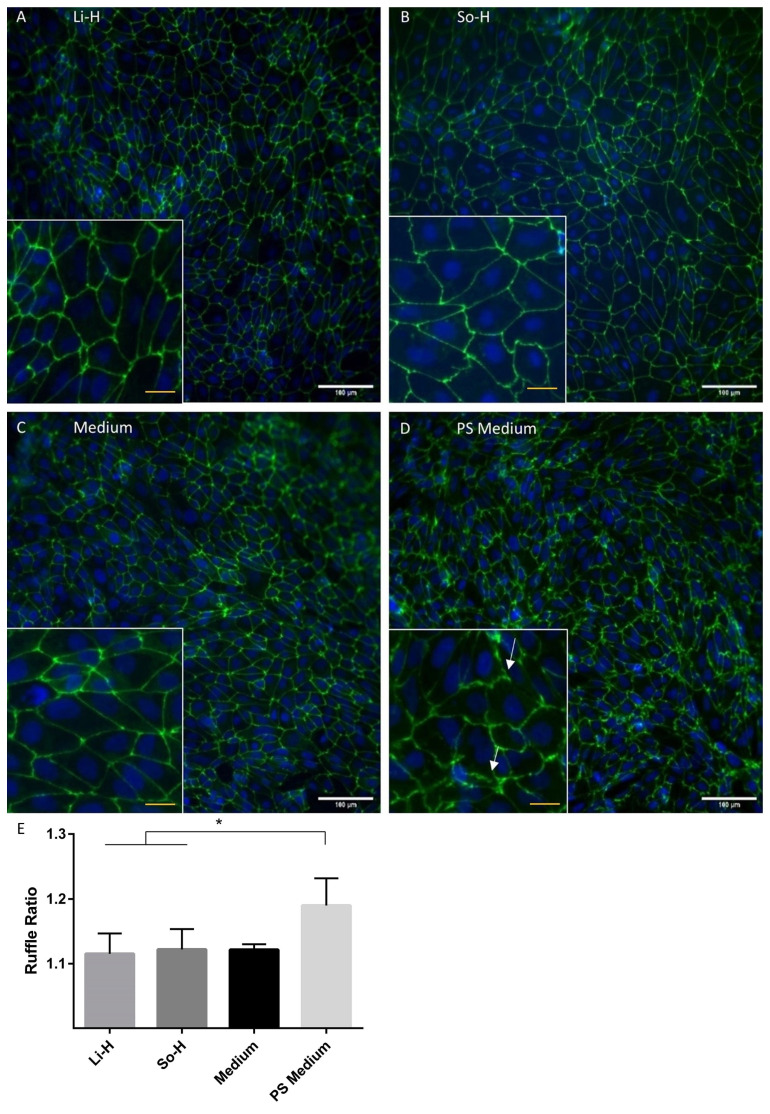
ZO-1 (green) and DAPI (blue) staining of BMEC-like cells treated with (**A**) So-H and (**B**) Li-H plasma or (**C**) serum-free medium (ECM −/−) and (**D**) PS medium for 24 h. White arrows indicate PS triggered ruffles of ZO-1 staining. White scale bar 100 µm, yellow scale bar 25 µm. (**E**) Quantification of ruffles. N = 3. Statistics: paired *t*-test, * *p* < 0.05.

For morphological and continuity analysis of the cell–cell contacts, a ZO-1 staining was performed. Whereas the plasma-treated groups were structurally comparable to the medium control group with continuous straight lines, the PS treatment resulted in more ruffles and unsteady lines. The quantification proved a significant increase in the ruffle ratio by the application of PS. However, no significant larger blank areas or missing cell–cell contacts were observed for any of the treatments (Figure 6).

### 2.4. Characterization of Astrocyte and Pericyte Differentiation

**Figure 7:**
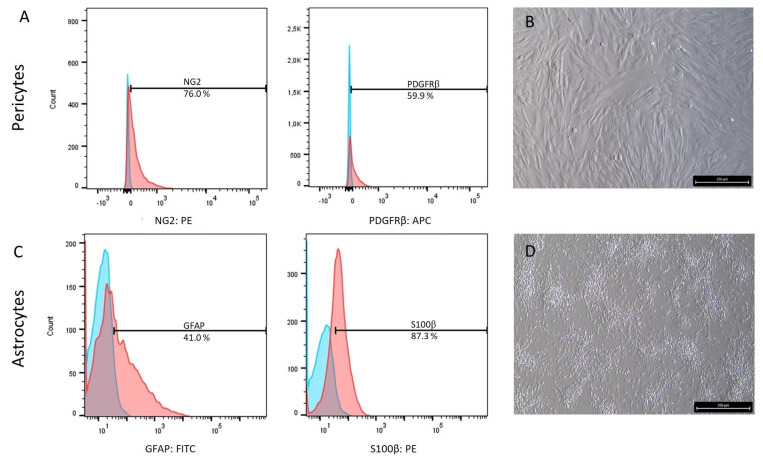
(**A**,**C**) Cellular markers and (**B**,**D**) phase contrast microscopy images for the evaluation of (**A**,**B**) pericyte and (**C**,**D**) astrocyte differentiation. Isotype antibody control is depicted in blue and stained samples in red. Scale bar 250 µm.

In order to also test plasma compatibility for multicellular models, we established an isogenic triple-culture model of the BBB. Here, IMR90-4 cells were additionally differentiated into astrocytes and pericytes. For each of the two cell types, two specific markers were determined to evaluate the differentiation outcome. Regarding pericytes, both markers were sufficiently expressed after differentiation with 76.0% NG2 and 59.9% PDGFRβ positive cells, whereas astrocytes reached 41.0% GFAP and 87.3% S100β positive cells (Figure 7). Consequently, both differentiated cell types were used for long-term analysis of the plasma compatibility in a triple-culture BBB model.

### 2.5. Gradual Assembly of the Coculture Model Confirms Positive Impact of Plasma Incubation and Coculture with Pericytes and Astrocytes on the Barrier Integrity of BMEC-like Cells

**Figure 8:**
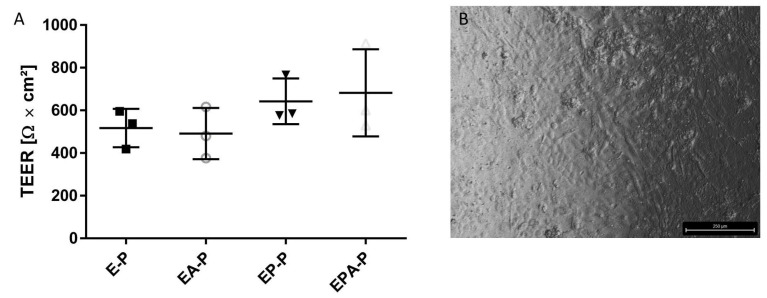
(**A**) TEER analysis of gradually assembled triple-culture BBB model with BMEC-like cells (E), pericytes (P), and astrocytes (A) incubated in plasma (-P) for 24 h. (**B**) Phase contrast microscopy of the BMEC-like cell layer in a triple-culture setup incubated for 72 h with So-H plasma. Scale bar 250 µm. N = 3.

Gradual assembly of the triple culture revealed positive reinforcement of the barrier integrity by coculturing pericytes and astrocytes with BMEC-like cells. Consequently, TEER could be enhanced from 517.1 ± 90.1 Ω × cm^2^ for the monoculture of BMEC-like cells to 681.8 ± 204.2 Ω × cm^2^ for the triple culture. Additional morphological analysis revealed an intact endothelial monolayer of BMEC-like cells after 72 h of incubation with plasma in a triple-culture setting (Figure 8).

### 2.6. Continuous TEER Analysis Reveal Regenerative Effect of Coculture Under Plasma Treatment

**Figure 9:**
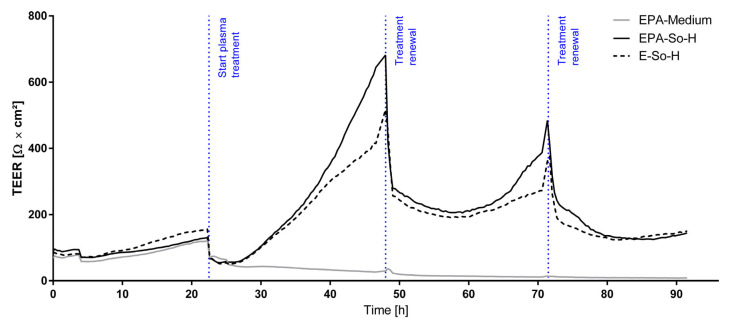
Continuous TEER measurements of triple culture (EPA-So-H, solid black line) or BMEC-like monoculture (E-So-H, dashed black line) treated with So-H compared to triple culture treated with serum-free ECM −/− medium (EPA-Medium, solid grey line). N = 3.

By continuous TEER analysis under the influence of plasma, initial barrier weakening could be observed after each treatment with plasma. With each treatment renewal, this phase of barrier weakening elongated. Whereas the phase of barrier integrity reduction had already changed 2 h after treatment initiation with plasma into barrier tightening, the same phase lasted for more than 10 h after the first treatment renewal for the triple culture model. After the second treatment renewal, the regenerative effect was noticeably attenuated. In general, this effect of barrier regeneration after initial weakening is only observed in triple culture models over the full treatment duration, whereas monocultures of BMEC-like cells showed this effect only after treatment initiation but not after the first treatment renewal. Medium-treated models did not exhibit any of the aforementioned effects observed in plasma-treated models. Instead, they demonstrated a consistent decrement in TEER over time, ultimately resulting in TEER values approaching zero (Figure 9).

## 3. Discussion

hiPSC-based BBB models have emerged in recent years as a promising tool for studying complex pathologies or drug development. Although these kinds of models have many advantages over in vivo animal experiments regarding comparability and translational potential towards human physiology, most models fail to reassemble the physiological complexity of the BBB to the full extent. The most widely used differentiation protocol for the generation of BMEC-like cells, published by Lippmann et al. [20], was proven by Lu et al. [15] to produce cells exhibiting both endothelial and epithelial characteristics. This study aims at contributing to more physiological BBB models by the application of human plasma as external stimulus. In this way, not only was a more physiological phenotype of the differentiated BMEC-like cells supposed to be established but also the potential use of pathological human plasma for the induction of pathological processes in in vitro BBB models.

BMEC-like cells differentiated in this study confirmed the lack of some endothelial cell markers as already described by Lu et al. [15] and Delsing et al. [14]. In detail, GLUT1 and ZO-1 were very well expressed in over 90% of the differentiated cells, and the endothelial tube formation assay indicated endothelial behavior, although VECad, vWF, and CD31 expression were very low in the FACS analysis. Although a lack of VECad and CD31 was also reported by Lu et al. [15] and Delsing et al. [14], the original protocol by Lippmann et al. [20] as well as the further developed protocol by Appelt-Menzel et al. [21] showed consistent VECad and CD31 expression in differentiated cells. Regarding vWF expression in this study, a small shift in fluorescence intensity could be detected by FACS analysis, indicating a very low basal expression as already stated by Delsing et al. [14]. Interestingly, the application of So-H plasma on differentiated BMEC-like cells induced an intracellular signal of vWF near the nucleus, which indicates a beneficial impact of plasma on the endothelial phenotype of differentiated BMEC-like cells. This observation was also reported in a study by Zhang et al., where an improvement of endothelial differentiation of adipose-derived stem cells by the application of 2% autologous plasma during differentiation could be shown in terms of higher vWF and CD31 expression compared to FBS-supplemented differentiation medium [22]. Consequently, the differentiated BMEC-like cells in this study confirm the discussion about the lack of endothelial cell markers after differentiation, although the application of So-H plasma seems to be a potential improvement for this issue. Therefore, these cells were used for further experiments to evaluate plasma compatibility.

We then evaluated the compatibility of BMEC-like cells with different human plasmas and serum in terms of basic parameters such as viability and LDH secretion. For clinical diagnostics, a variety of different anticoagulated plasmas were established over time, whereas the choice of anticoagulation is dependent on the stability and measurability of the analyte [23]. While EDTA and citrate plasmas, as well as serum, significantly impaired cell viability compared to the medium control group, heparin-anticoagulated plasmas showed no adverse effects. This can be attributed to the different modes of action of the anticoagulants. EDTA and citrate plasmas chelate calcium, which thereby induce hypocalcemia and consequently interrupt the coagulation cascade [24,25,26]. On the other hand, heparin induces increased activity of native antithrombin without changing the concentration of plasma contents to such an extent as EDTA and citrate [27,28]. Consequently, the observed negative effects of citrate and EDTA anticoagulated plasmas can be explained by the lack of calcium, which is fundamental for cell adhesion and keeping homeostasis in the cell culture [29,30,31]. More specifically, low extracellular calcium levels were shown to induce an inflammatory phenotype and a decline in cell–cell contacts leading to severe impairment of the barrier function of BBB-specific microvascular endothelial cells [32,33]. Pathologically, hypocalcemia is associated with a variety of neuromuscular symptoms like seizures or numbness, underlining the important role of calcium in physiological processes [34]. However, EDTA anticoagulated plasmas induced more severe effects as they are stronger chelation reagents than citrate [35]. Therefore, not only calcium but also other metals like manganese, magnesium, and iron are chelated more effectively with ETDA than with citrate, which can lead to additional ROS accumulation and lipid metabolism disorders in vascular endothelial cells [36,37,38].

On the other hand, serum also induced significant deterioration of viability and LDH secretion compared to the medium control group. This can be attributed to alterations in fibrinogen, potassium, calcium, and general protein content compared to heparinized plasma due to the coagulation process [23,39,40]. Additionally, it has been demonstrated that IL-8 secretion is induced by coagulation, which activates NO signaling pathways, leading to increased permeability and the internalization of VE cadherin of endothelial cells [41].

Based on these results, Li-H and So-H plasmas were determined to be the most compatible amongst the tested plasma and serum samples. Therefore, these two candidates were further evaluated regarding their impact on the barrier function of BMEC-like cells. Here, significant improvements in the barrier integrity could be shown by TEER and FITC-dextran permeability assessments, although no significant difference in cell density could be detected in comparison to the medium control group. Consequently, a strengthening of barrier functionality by improvement of the cell–cell contacts can be assumed. Similar results upon plasma treatment of endothelial cells were reported by Gallagher et al., where the electrical resistance of HUVEC could already be increased after 15 min of incubation with 10% plasma in medium compared to the medium control group [42]. Additionally, the pathological reaction of BMEC-like cells to PS in medium as well as in plasma could be shown by TEER and FITC-dextran analysis as well as cell density measurements. The components of the PS were selected based on their relevance to the pathology of sepsis, which serves as a prime example of a systemic inflammatory condition affecting the BBB. The pathology is characterized by significant accumulation of cytokines and pro-inflammatory stimuli within the blood compartment, which is often neglected in pathological in vitro models of the BBB [43]. This could be improved by the use of pathological plasma in the future. The concentrations of the PS components in medium were oriented towards reported maximum concentrations of septic patients, where for LPS 5.1 ng/mL [44], IL-6 2.0 ng/mL [45], TNF-α 1.5 ng/mL, and IL-1β 1.9 ng/mL [46] were reported. However, much higher concentrations needed to be applied in order to elicit a measurable barrier breakdown after 24 h of incubation (Supplementary File, Figures S1 and S2). Furthermore, PS in plasma had to be applied 10× higher than in medium due to protein–protein interactions and consequently less bioavailability. As already shown by Rosenberg-Hasson et al., the recovery rate of cytokine spiked plasmas can be limited to about 87.8% IL-1β, 22.5% IL-6, and 58.9% TNF-α in multiplex immunoassays due to plasma matrix effects [47]. These effects include cytokine–protein interactions, whereas binding partners can be proteins like albumin or fibrinogen [48]. This can lead to an ameliorated pathological effect of spiked PS in vitro as already shown by Duran-Guell et al. [49]. Consequently, the necessary increase in PS concentration in plasma can be explained by plasma matrix effects rather than a decreased responsiveness of BMEC-like cells.

Finally, plasma compatibility was evaluated in an isogenic triple-culture BBB model with BMEC-like cells, astrocytes, and pericytes. Here, the beneficial effects of coculturing pericytes and astrocytes with BMEC-like cells could be observed, whereas pericytes induced higher barrier strengthening than astrocytes. These results confirm observations reported by several studies, where pericytes improved the barrier integrity of BMEC-like cells and induced higher effects than cocultures with astrocytes [13,50]. Additionally, plasma application on the gradually assembled triple culture did not show any negative effects on the barrier integrity. In fact, the model was consistently improved by plasma incubation, thereby confirming the positive impact of plasma in vitro. However, in comparison with other studies, which reported TEER values of about 2300 Ω × cm^2^ for BMEC-like monocultures, the TEER values for the triple-culture model in this study were rather low [50]. This can be attributed to the coculture medium on the basolateral side, which is not optimized for BMEC-like cells and consequently has an inhibitory effect on the barrier formation.

Continuous TEER analysis revealed an initial decrease upon plasma treatment and treatment renewal, which can be explained by a harsh change in the extracellular environment and the following time for cell adjustment [51]. This initial barrier weakening was also reported by Gallagher et al., who observed this effect already by an application of 10% plasma [42]. However, the initial weakening changed after a few hours to barrier tightening and improvement of the BMEC-like cell functionality compared to the medium control group. Here, the triple culture showed higher regenerative potential than the BMEC-like monoculture. This can be explained by positive reinforcement of the three cell types used in the model. Thus, pericytes are known to secrete angiogenic stimuli like VEGF and can stimulate the production of extracellular matrix molecules like laminin and fibronectin, consequently contributing to barrier integrity improvement [2,52,53,54]. On the other hand, a lack of pericytes is physiologically associated with poor vascular stability and deficient capillary density [2,54,55]. Regarding astrocytes, it is known that they can strengthen endothelial tight-junction proteins and induce endothelial polarity, leading to increased barrier functionality [56,57]. Consequently, it can be concluded that the positive impact of plasma on the barrier function of BMEC-like cells can be also observed in triple-culture models, and the beneficial physiological interactions of the three cell types are not disturbed by the plasma application.

## 4. Materials and Methods

**Figure 10:**
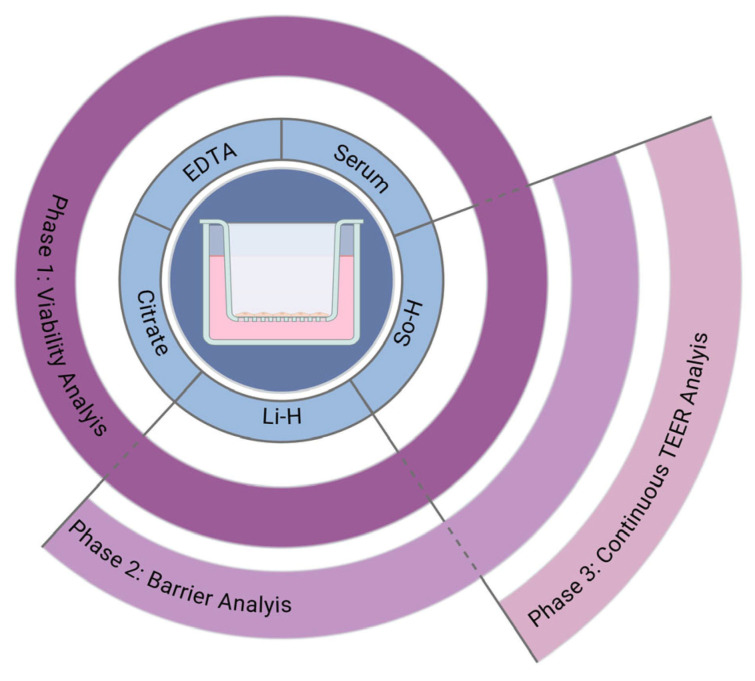
Schematical overview of the experimental design of this study. Sodium heparin (So-H), lithium heparin (Li-H), and ethylenediaminetetraacetic acid (EDTA) [58].

This study was performed in three phases, as depicted in Figure 10. In phase 1, the effect of different plasmas and serum on differentiated BMEC-like cells regarding basic parameters like LDH secretion and viability was evaluated after 24 h of treatment. Subsequently, the barrier-forming properties of BMEC-like cells incubated with So-H or Li-H plasma for 24 h were characterized in phase 2. In phase 3, continuous TEER analysis of an isogenic triple-culture model of the BBB, which was incubated with So-H plasma for 72 h, was conducted.

### 4.1. Human Plasma Pool

Human plasma was collected from three healthy donors (without permanent medication) according to standard operating procedures at the University Medical Center, Rostock. In short, blood samples were drawn in sodium-heparin (So-H), lithium-heparin (Li-H), citrate, ethylenediaminetetraacetic acid (EDTA), and serum Monovettes (Sarstedt, Nümbrecht, Germany). Samples were centrifuged at 3220× *g* for 10 min at 4 °C. The resulting plasma was pooled and stored at −80 °C until it was used for experiments.

### 4.2. Cell Culture

#### 4.2.1. Immortalized Human Cerebral Microvascular Endothelial Cell Line D3 (hCMEC/D3)

The immortalized cell line hCMEC/D3 was used as a positive control for the endothelial tube formation assay and the establishment of the PS. Cells were cultured on cell+ cell culture flasks (Sarstedt, Germany) in endothelial cell growth medium (ECGM) (PromoCell, Heidelberg, Germany).

#### 4.2.2. Brain Microvascular Endothelial Cell-like Cells (BMEC-like Cells)

The hiPSC line IMR90-4 (WiCell Reasearch Institute, Madison, WI, USA) was cultured on growth factor reduced Matrigel-coated (Corning, Corning, NY, USA) 6-well plates in mTeSR plus medium (Stemcell Technologies, Vancouver, BC, Canada) at 37 °C, 5% CO_2_, and 95% humidity. At 60% confluency, cells were passaged with Accutase (PAN Biotech, Aidenbach, Germany) and seeded for differentiation at a density of 7500 cells/cm^2^. Differentiation was performed with minor modifications according to a protocol published by Lippmann et al. and Haferkamp et al. [20,50]. In short, two days after seeding, medium was switched to unconditioned medium composed of DMEM/F12 supplemented with 20% knockout serum replacement, 0.1 mM mercaptoethanol, 1 mM L-Glutamine, and 1% MEM non-essential amino acids (Thermo Fisher Scientific, Waltham, MA, USA). Afterwards, daily medium changes with UM were performed for five consecutive days. On day 6, medium was switched to serum-free EC medium (ECM +/+) (human endothelial serum-free medium supplemented with 0.5% B27 Supplement (Thermo Fisher Scientific, USA), 10 µM retinoic acid (Sigma-Aldrich, St. Louis, MO, USA), and 20 ng/mL human basic FGF (Thermo Fisher Scientific, USA)). On day 8, cells were harvested using Accutase and seeded onto collagen IV/fibronectin (40% collagen (1 mg/mL) and 20% fibronectin (0.5 mg/mL) (Sigma-Aldrich, USA) in distilled water and incubated overnight at 37 °C) coated polyester tissue culture (TC) inserts in serum-free ECM +/+ (0.33 cm^2^, pore size 0.4 µm, transparent) (Sarstedt) at a density of 1 × 10^6^ cells/cm^2^. The next day, medium was switched to human endothelial serum-free medium supplemented with 0.5% B27 Supplement without human basic FGF and retinoic acid (ECM −/−).

#### 4.2.3. Astrocytes and Pericytes

HiPSC-derived astrocytes were differentiated from IMR90-4 using the embryoid body protocol by Stemcell Technologies [59]. All steps were performed according to the manufacturer’s protocol.

For hiPSC-derived pericytes, IMR90-4 cells were differentiated into neural crest cells by using the STEMdiff neural crest differentiation kit (Stemcell Technologies, Canada) [60]. Resulting cells were further differentiated, inspired by Gastfriend et al., by seeding onto Matrigel-coated T25 flasks in TeSR E6 medium (Stemcell Technologies, Canada) supplemented with 10% fetal bovine serum (FBS) (Thermo Fisher Scientific, USA) [61]. As soon as the cells were at about 90% confluence, cells were passaged onto an uncoated cell+ cell culture flask (Sarstedt, Germany). As soon as the cells reached 90% confluency again, cells were passaged onto an uncoated standard cell culture flask. At this point, cells were considered to be mature and were used for experiments.

#### 4.2.4. Establishment of a Triple-Culture BBB Model

For the coculture of BMEC-like cells, astrocytes, and pericytes, TC inserts were coated on the apical side with collagen/fibronectin as described above. The following day, the TC inserts were flipped upside down and coated on the basolateral side again with collagen and fibronectin as already described, before incubation for 1 h at room temperature. Afterwards, pericytes and astrocytes were passaged, and 30,000 astrocytes and 43,000 pericytes per TC insert were seeded in coculture medium onto the basolateral side. Cells were allowed to attach onto the membrane for 1 h at room temperature. Then, the TC inserts were again flipped, and BMEC-like cells (day 8) were seeded in ECM +/+ at a density of 1 × 10^6^ cells/cm^2^ on the apical side. Volumes were adjusted to 200 µL of ECM +/+ on the apical side and 500 µL of coculture medium on the basolateral side. Serum-free coculture medium was composed as described by Stemcell Technologies (2% NeuroCult SM1, 1% N2 Supplement-A, 20 ng/mL BDNF, 20 ng/mL GDNF, 1 mM Dibutyryl-cAMP, 200 nM ascorbic acid, and 0.4% STEMdiff microglia supplement 2 in BrainPhys Neuronal Medium (Stemcell Technologies, Canada)) [62]. After 24 h, the apical medium was switched to ECM −/−. The next day, plasma treatment was initiated (D0) and renewed every 24 h. With the beginning of the apical treatment and every consecutive medium change on the apical side, a half medium change on the basolateral side was performed with coculture medium.

### 4.3. Immunofluorescence Staining and Fluorescence-Activated Cell Sorting (FACS) Analysis

All incubation steps were performed at room temperature on a rocking shaker (30 rpm) unless otherwise stated. Cells were fixed for 10 min with 4% freshly prepared PFA solution (Thermo Fisher Scientific, USA) at room temperature and permeabilized by applying 0.2% TWEEN20 (Carl Roth, Karlsruhe, Germany) in PBS for 15 min at 4 °C. Non-specific binding was blocked by an incubation with bovine serum albumin (Carl Roth, Germany) (5% in PBS) for 30 min before incubation with the primary antibody ZO-1 (Thermo Fisher Scientific, USA) (1:200) or vWF (Thermo Fisher Scientific, USA) (1:50) for 60 min. Subsequently, incubation with the secondary antibody goat anti-mouse 488 (Thermo Fisher Scientific, USA) (1:200) was performed for 60 min. Finally, cells were counterstained with DAPI (Thermo Fisher Scientific, USA) (300 nM in PBS) for 5 min and analyzed with the fluorescence microscope Eclipse Ti (Nikon, Minato, Tokio, Japan).

For FACS analysis, cells were detached from the well using Accutase and stained as already described before with GLUT1 (Thermo Fisher Scientific, USA) (1:100), ZO-1 (Thermo Fisher Scientific, USA) (1:200), vWF (Thermo Fisher Scientific, USA) (1:50), VECad (Miltenyi Biotec, Bergisch Gladbach, Germany) (1:50), CD31 (Miltenyi Biotec, Germany) (1:50), GFAP (Miltenyi Biotec, Germany) (1:50), S100β (Miltenyi Biotec, Germany) (1:50), NG2 (Miltenyi Biotec, Germany) (1:50), and PDGFRβ (Miltenyi Biotec, Germany) (1:50). For each staining, 10,000 cells were recorded. Measurement was performed using a BD FACS Verse (Becton Dickinson, Franklin Lakes, NJ, USA). Data analysis was performed with FlowJo 10.10.0 (Becton Dickinson, USA). Before determination of percentual frequencies, debris and doublets were excluded using forward and side scattering.

### 4.4. Endothelial Tube Formation Assay

BMEC-like cells (day 8) were seeded onto collagen/fibronectin-coated well plates in ECM +/+. After 24 h, cells were detached from the well bottom with Accutase, and 10,000 BMEC-like cells or hCMEC/D3 were seeded onto an angiogenesis glass bottom µ-slide (ibidi, Gräfelfing, Germany) in ECM +/+ (BMEC-like cells) or ECGM (hCMEC/D3) supplemented with 40 ng/mL VEGF. The specialized µ-slide was filled beforehand with 10 µL/well growth factor-reduced Matrigel and incubated for 1 h at 37 °C. The slide was placed in the Nikon Eclipse Ti live cell imaging chamber (Nikon, Japan) and monitored for 3 h.

### 4.5. Plasma and Inflammatory Treatment for Viability, LDH Secretion, and Barrier Analysis

Treatment of BMEC-like cells was performed one day after differentiated BMEC-like cells (day 8) were seeded onto collagen/fibronectin-coated well plates. Plasma and serum treatment was applied for 24 h at concentrations of 100%, 75%, and 50% diluted in serum-free ECM −/−. Combined sepsis-associated stimuli are referred to as pathological stimulation (PS) and consisted of 50 ng/mL TNFα, 100 ng/mL IL-6, IL-1β (BioLegend, San Diego, CA, USA), and 10 µg/mL lipopolysaccharide (LPS) (Sigma-Aldrich, USA) in cell culture medium. PS in plasma was prepared at 10× concentration.

### 4.6. Viability Analysis and Lactate Dehydrogenase Secretion

After treatment, the supernatant was collected and centrifuged for 5 min at 200× *g*. The resulting supernatant was analyzed regarding lactate dehydrogenase (LDH) content using a Cobas Mira Plus (Roche, Basel, Switzerland). Adherent cells were harvested with Accutase and pooled with the respective cell pellet from the supernatant samples. Afterwards, cells were stained with DAPI (Miltenyi Biotec, Germany) (1:200 in PBS). The viability was measured by quantifying the proportion of DAPI unstained (live) and stained (dead) cells with the MACS Quant Analyzer 16 (Miltenyi Biotec, Germany).

### 4.7. Barrier Functionality Analysis

#### 4.7.1. Transendothelial Electrical Resistance

Transendothelial electrical resistance (TEER) of BMEC-like cells was measured after treatment using a chopstick MERSSTX03 electrode (Merck Millipore, Billerica, MA, USA). Before measurement, a medium change to fresh, room temperature, cell culture medium was performed, and the electrode was allowed to equilibrate for 15 min in the respective medium. Each TC insert was measured at three positions. Empty collagen IV/fibronectin-coated TC inserts served as blanks. Continuous TEER measurements were performed using a CellZScope+ impedance spectrometer (nanoAnalytics, Münster, Germany) in a range of 1 Hz to 100 kHz with a waiting time of 15 min between each measurement.

#### 4.7.2. FITC-Dextran Permeability

Permeability across the endothelial cell layer was assessed by applying a 4 kDa FITC-dextran solution (Sigma-Aldrich, USA) at a concentration of 10 µg/mL in the respective cell culture medium on the apical side of the TC inserts. After 1 h of incubation at 37 °C, samples were taken from the basolateral side and measured for the FITC-specific fluorescence intensity with a plate reader (ClarioStar, BMG Labtech, Ortenberg, Germany). Based on these results, the permeability coefficient was calculated according to Siflinger-Birnboim et al. [63].

### 4.8. Image Analysis

Fiji 1.54g [64] was used for quantifying the ruffle ratio as described by Tokuda et al. [65]. For automated cell density quantification, QuPath version 0.3.2 was used [66].

### 4.9. Statistical Analysis

Statistical analysis was conducted using GraphPad Prism version 6.07 (GraphPad Software, Boston, MA, USA). Normal distribution of the data was tested with D’Agostino and Pearson omnibus normality test, or assumed for N too small. *p*-value ≤ 0.05 was considered significant. Indicated values are presented as mean ± standard deviation. N refers to biological replicates.

## 5. Conclusions

Undiluted heparin plasmas were found to be compatible with BMEC-like cells without altering viability after 24 h of incubation. Although differentiated BMEC-like cells showed good barrier characteristics, they also confirmed poor expression of some specific endothelial markers like VECad, vWF, and CD31. However, vWF expression could be improved by the application of So-H plasma, indicating a positive effect of plasma on the endothelial phenotype of differentiated BMEC-like cells. Additionally, barrier integrity could be significantly increased by the application of Li-H and So-H plasmas without altering cell density, which indicates a strengthening of cell–cell contacts upon plasma treatment. Finally, plasma application was evaluated in an isogenic triple-culture model of the BBB with BMEC-like cells, astrocytes, and pericytes. Here, the aforementioned beneficial impact of plasma could be confirmed. Thus, the application of undiluted heparinized plasma in in vitro BBB models can be considered beneficial for the endothelial characteristics of differentiated BMEC-like cells. Consequently, these results pave the way for using pathologically conditioned plasmas to generate pathological BBB models, which will further improve the translational potential of in vitro BBB models.

## Data Availability

Research data are available on request from the corresponding author.

## Supplementary Materials

The following supporting information can be downloaded at: https://www.mdpi.com/article/10.3390/ijms26073334/s1. Reference [67] is cited in the supplementary materials.

## Abbreviations

The following abbreviations are used in this manuscript:

BBB	Blood–brain barrier
BMEC	Brain microvascular endothelial cells
CNS	Central nervous system
DAPI	4′,6-diamidino-2-phenylindole
ECM +/+	Endothelial culture medium with FGF and retinoic acid
ECM −/−	Endothelial culture medium without FGF and retinoic acid
ECGM	Endothelial cell growth medium
EDTA	Ethylenediaminetetraacetic acid
FACS	Fluorescence-activated cell sorting
FBS	Fetal bovine serum
FGF	Fibroblastic growth factor
GFAP	Glial fibrillary acidic protein
GLUT1	Glucose transporter 1
hiPSC	Human induced pluripotent stem cells
IL-1β	Interleukin-1β
IL-6	Interleukin-6
LDH	Lactate dehydrogenase
Li-H	Lithium heparin
LPS	Lipopolysaccharide
NG2	Neural/glial antigen 2
PDGFRβ	Platelet-derived growth factor receptor β
PS	Pathological stimulation
S100β	S100 calcium binding protein β
So-H	Sodium heparin
TC	Tissue culture
TNF-α	Tumor necrosis factor α
TEER	Transendothelial electrical resistance
VECad	Vascular endothelial cadherin
VEGF	Vascular endothelial growth factor
vWF	Von Willebrand factor
ZO-1	Zonula Occludens-1
