# Supplementary material for: Ex Vivo Plasma Application on Human Brain Microvascular Endothelial-like Cells for Blood–Brain Barrier Modeling
Source: Int J Mol Sci. 2025 Apr 3;26(7):3334. doi: 10.3390/ijms26073334 (PMC11989380; doi:10.3390/ijms26073334)
Supplement: Supplementary file 1 [file ijms-26-03334-s001.zip › ijms-3458422-supplementary.pdf]

Ex Vivo Plasma Application on Human Brain Microvascular Endothelial-like Cells for Blood–Brain Barrier Modeling

Supplementary File

Establishment of the PS

Methods

Individual Cytokine/LPS stimulation

Pathological stimuli IL-6, IL-1β, TNFα (100 ng/ml each) and LPS (10 μg/ml) in ECGM were applied on hCMEC/D3 ($3.45*10^4$ cells/cm^2) for 24 h and 72 h. Afterwards, LDH secretion and viability was quantified as described in the main paper.

Combined Cytokine/LPS stimulation (PS)

hCMEC/D3 were seeded with a density of $3.45*10^4$ cells/cm^2 and BMEC-like cells with 1.0×10^6 cells/cm^2 on collagen IV/ fibronectin coated TC-inserts. After 24 h, cells were treated with 100 ng/ml IL-6, 100 ng/ml IL-1β, 50 ng/ml TNFα and 10 μg/ml LPS in ECGM for hCMEC/D3 or ECM -/- for 24 h. Afterwards, TEER and cell density were quantified as described in the main paper.

Results

Aim of the establishment of the PS was to keep good viability of the cells, while breaking down barrier formation. First, individual cytokines and LPS were applied for 24 h treatment, which induced no adverse effects regarding viability and LDH secretion. Consequently, incubation was prolonged to 72 h. Thus, more distinct effects of the stimuli could be observed. Therefore, especially TNFα induced as the only stimulus a viability decrease from 89.8 ± 2.3 % in medium to 85.4 ± 3.5 %. Furthermore, LDH secretion was significantly increased by the application of TNFα for 72 h (11.7 ± 2.8 U/l) compared to the medium control group (5.6 ± 0.5 U/l). Other stimuli did not show strong effects on the viability and LDH secretion. As a result, we decided to halve the concentration of TNFα compared to the other stimuli for the generation of the PS (Figure S1).

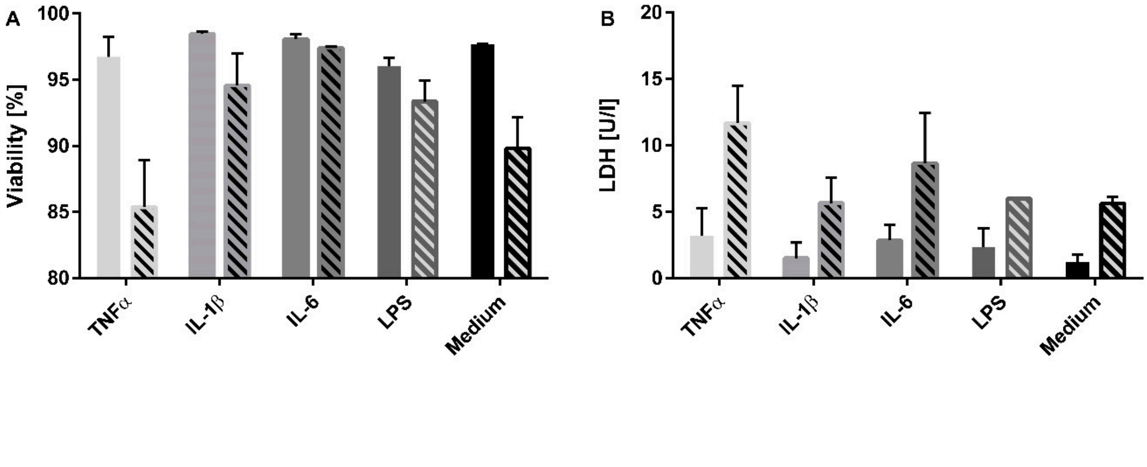

Figure S1: (A) viability and (B) LDH secretion of hCMEC/D3 cells treated for 24 h (left) and 72 h (right, dashed) with 100 ng/ml TNFα, IL-1B, IL-6 and 10 µg/ml LPS compared to ECGM medium. N = 2

PS was applied for 24 h before TEER and cell density measurements. For both cell types significant decrease of TEER could be detected for BMEC and hCMEC by the application of PS. Therefore, TEER of BMECs decreased from 521.7 ± 472.4 Ω*cm^2 to 138.6 ± 168.3 Ω×cm^2 and of hCMEC/D3 from 20.4 ± 5.6 Ω×cm^2 to 10.5 ± 2.3 Ω×cm^2 with PS. Generally, hCMEC TEER was quite low compared to the BMEC TEER in medium as expected from literature [67].

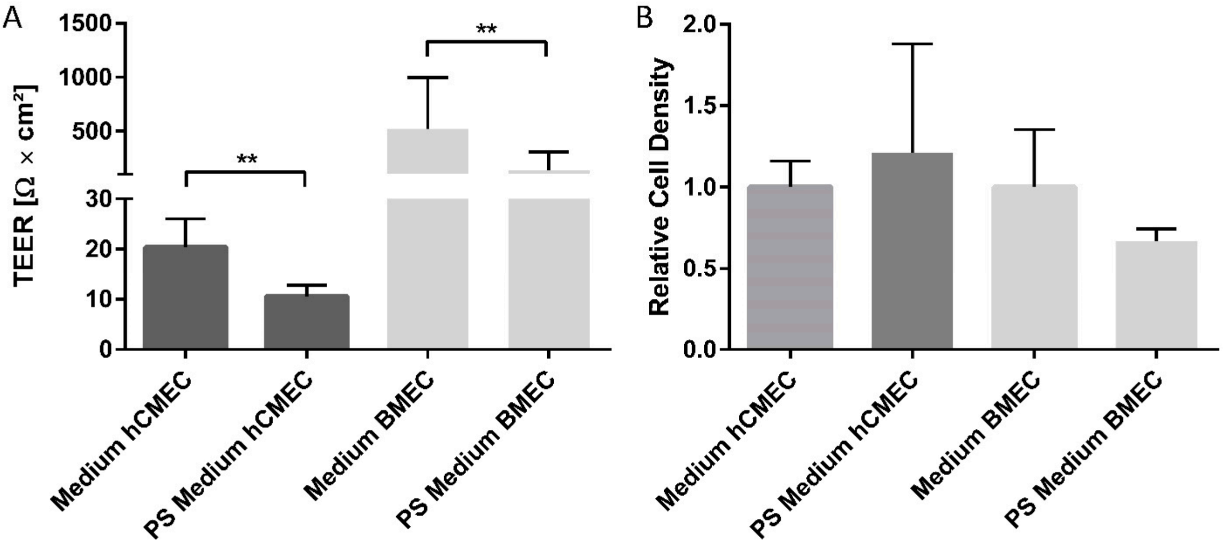

Figure S2: (A) TEER measurements and (B) relative cell density of hCMEC/D3 and BMEC-like cells treated with PS in the respective serum-free medium (ECGM/ECM -/-) compared to the serum-free medium control group. TEER: N = 8 for BMEC and N = 4 for hCMEC/D3. Cell density: N = 3 for both. Statistics: ratio paired t-test ** $p < 0.01$

References

67. Weksler, B.; Romero, I.A.; Couraud, P.-O. The hCMEC/D3 cell line as a model of the human blood brain barrier. *Fluids Barriers CNS* **2013**, *10*, 16, doi:10.1186/2045-8118-10-16.
